# Phoenixin 20 ameliorates pulmonary arterial hypertension via inhibiting inflammation and oxidative stress

**DOI:** 10.18632/aging.205468

**Published:** 2024-03-19

**Authors:** Yaqin Chai, Xing Gu, HongJun Zhang, Xinting Xu, Lizhan Chen

**Affiliations:** 1 Department of Pulmonary and Critical Care Medicine, Xi’an Chest Hospital, Xi’an 710100, China; 2 Department of Pulmonary and Critical Care Medicine, Xi’an International Medical Center Hospital, Xi’an 710100, China

**Keywords:** Phoenixin 20, pulmonary arterial hypertension, oxidative stress, NLRP3, SIRT1

## Abstract

Pulmonary arterial hypertension (PAH) is a severe pathophysiological syndrome resulting in heart failure, which is found to be induced by pulmonary vascular remodeling mediated by oxidative stress (OS) and inflammation. Phoenixin-20 (PNX-20) is a reproductive peptide first discovered in mice with potential suppressive properties against OS and inflammatory response. Our study will explore the possible therapeutic functions of PHN-20 against PAH for future clinical application. Rats were treated with normal saline, PHN-20 (100 ng/g body weight daily), hypoxia, hypoxia+PHN-20 (100 ng/g body weight daily), respectively. A signally elevated RVSP, mPAP, RV/LV + S, and W%, increased secretion of cytokines, enhanced malondialdehyde (MDA) level, repressed superoxide dismutase (SOD) activity, and activated NLRP3 signaling were observed in hypoxia-stimulated rats, which were notably reversed by PHN-20 administration. Pulmonary microvascular endothelial cells (PMECs) were treated with hypoxia with or without PHN-20 (10 and 20 nM). Marked elevation of inflammatory cytokine secretion, increased MDA level, repressed SOD activity, and activated NLRP3 signaling were observed in hypoxia-stimulated PMECs, accompanied by a downregulation of SIRT1. Furthermore, the repressive effect of PHN-20 on the domains-containing protein 3 (NLRP3) pathway in hypoxia-stimulated PMECs was abrogated by sirtuin1 (SIRT1) knockdown. Collectively, PHN-20 alleviated PAH via inhibiting OS and inflammation by mediating the transcriptional function of SIRT1.

## INTRODUCTION

Pulmonary arterial hypertension (PAH) is a group of pathophysiological syndromes with extremely complex pathogenesis. As pulmonary vascular resistance continues to increase, failure of the right side of the heart and death will inevitably progress, with an extremely poor prognosis [1]. In recent years, hypoxic pulmonary hypertension has become a research hotspot. The pathological process of PAH includes pulmonary vascular contraction and pulmonary vascular remodeling, and hypoxia is an important inducing factor in pulmonary vascular remodeling [2]. A previous study has shown that oxidative stress (OS) is a critical mechanism in the pathogenesis of PAH. After the formation of PAH, as the right ventricular lesions continue to progress, the right ventricle wall will gradually thicken. The thickened wall will markedly change the demand for oxygen, and the oxygen consumption will gradually increase, while the oxygen supply will gradually decrease, resulting in a reduction of the mechanical efficiency of the right ventricle [3, 4]. Meanwhile, excessive production of reactive oxygen species (ROS) in the body will be induced, causing damage to a variety of cellular macromolecules, thereby changing the transmission of cell signals and eventually causing cell apoptosis. As ROS is inactivated and removed after being catalyzed by superoxide dismutase (SOD), glutathione peroxidase (GSH-Px), and catalase (CAT), these enzymes can protect the body from free radical damage to a certain extent [5]. The decrease in SOD activity will lead to lipid peroxidation damage of the cell membrane thereby generating a large amount of malondialdehyde (MDA), which will cross-link with proteins and nucleic acids to destroy their normal structure and configuration. Eventually, the original characteristics and functions of proteins and nucleic acids will be affected, thus aggravating cell damage [6]. Furthermore, the inflammatory response also plays a vital role in PAH pathogenesis. In 1994, TUDER et al. [7] first demonstrated the presence of inflammatory infiltration in plexiform lesions in patients with PAH. Subsequently, COOL et al. [8] reported the presence of mononuclear inflammatory cells around plexiform vessels in PAH. Hence, controlling OS and inflammatory response may become the key direction for the PAH treatment.

Phoenixin (PNX) was originally identified as a novel reproductive peptide in mice due to its ability to bind to pituitary adenoma cell membranes. By inducing the expression of gonadotropin-releasing hormone receptor and increasing gonadotropin-releasing hormone, PNX stimulates luteinizing hormone (LH) release in pituitary cells of female mice [9]. Two active forms of PNX were discovered in mice, including PNX-14 and PHN-20 [10]. Chi [11] claimed that PHN-20 alleviated the pathological symptoms of gestational diabetes mellitus by repressing OS and inflammation. Sun reported the anti-inflammatory properties of PHN-20 in dental pulp cells [12]. However, the potential role of PHN-20 in PAH remains unknown. Our study will explore the possible therapeutic function of PHN-20 against PAH for future clinical application.

## RESULTS

### Treatment with PHN-20 attenuated hypoxia-induced hemodynamics in rats

**Figure 1:**
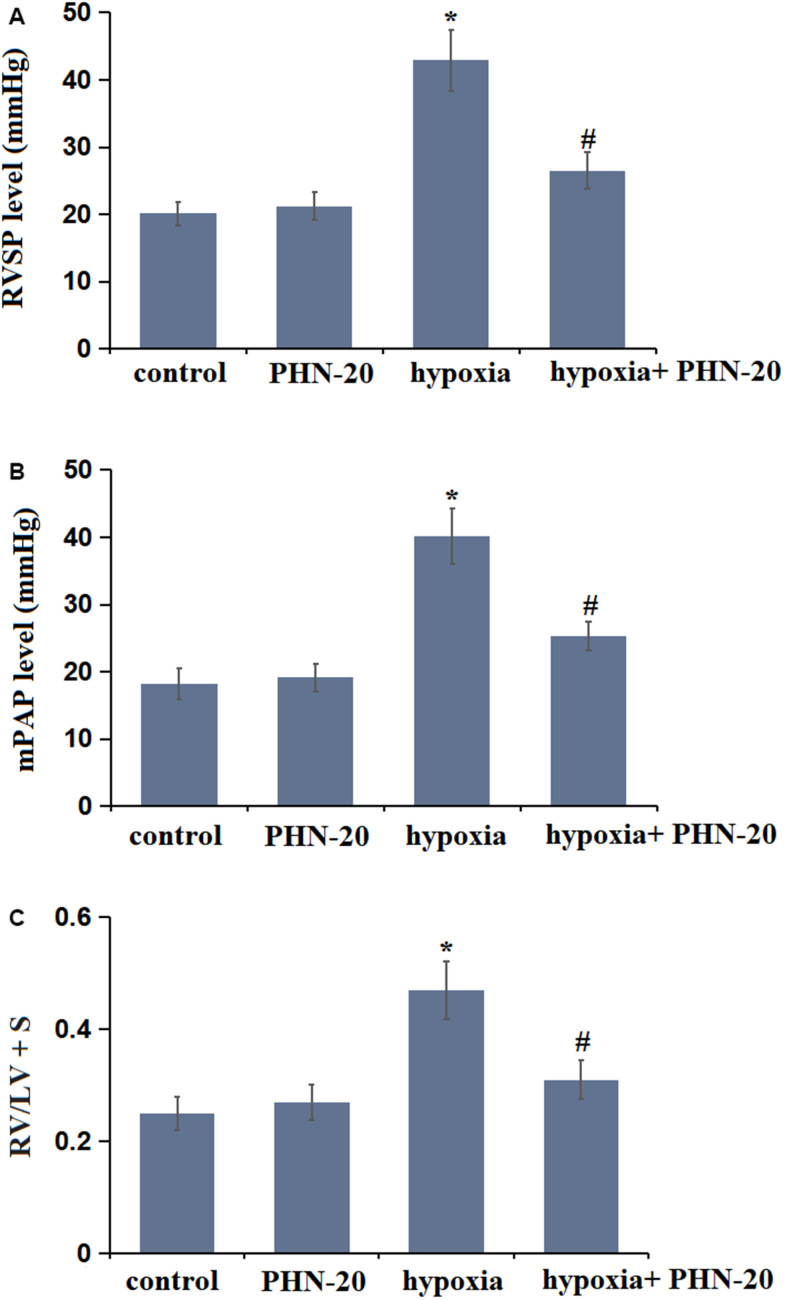
**Treatment with Phoenixin-20 (PHN-20) attenuated hypoxia-induced hemodynamics in rats.** (**A**) Right ventricular systolic pressure (RVSP) (mmHg), (**B**) mean pulmonary arterial pressure (mPAP) (mmHg), and (**C**) right ventricle-to-left ventricle+septum (RV/LV + S) were measured (*, P<0.05 vs. control group; #, P<0.05 vs. hypoxia group).

To evaluate the impact of PHN-20 on pulmonary arterial function, pulmonary hemodynamic parameters were measured. The RVSP value was maintained around 20 mmHg in the control and PHN-20 groups, while it was signally increased to 42.91 mmHg in the hypoxia group, then markedly repressed to 26.53 mmHg by PHN-20 (Figure 1A). Moreover, the mPAP values in the control, PHN-20, hypoxia, and hypoxia+ PHN-20 groups were 18.25, 19.17, 40.18, and 25.24 mmHg, respectively (Figure 1B). The value of RV/LV + S was kept at 0.25 and 0.27 in the control and PHN-20 groups, respectively, was extremely elevated to 0.47 by hypoxia, then notably reduced to 0.31 by PHN-20 (Figure 1C). Abnormal hemodynamics observed in hypoxia-treated rats were alleviated by PHN-20.

### Treatment with PHN-20 alleviated hypoxia-induced pulmonary vascular remodeling in rats

**Figure 2:**
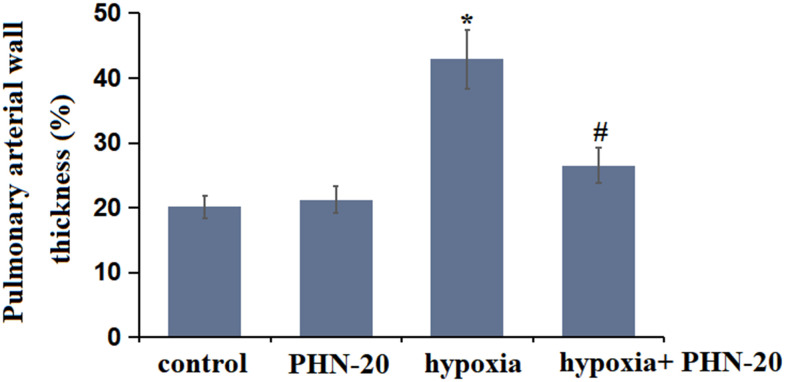
**Treatment with PHN-20 alleviated hypoxia-induced pulmonary vascular remodeling in rats.** The percentage of pulmonary arterial wall thickness (W%) was calculated. (*, P<0.05 vs. control group; #, P<0.05 vs. hypoxia group).

The W% in the control and PHN-20 groups was kept at 24.21% and 22.16%, respectively, was signally promoted to 45.71% by hypoxia then markedly decreased to 30.33% by PHN-20 (Figure 2), implying a protective property of PHN-20 on hypoxia-induced pulmonary vascular remodeling in rats.

### PHN-20 repressed inflammation in lung tissues of PAH rats

**Figure 3:**
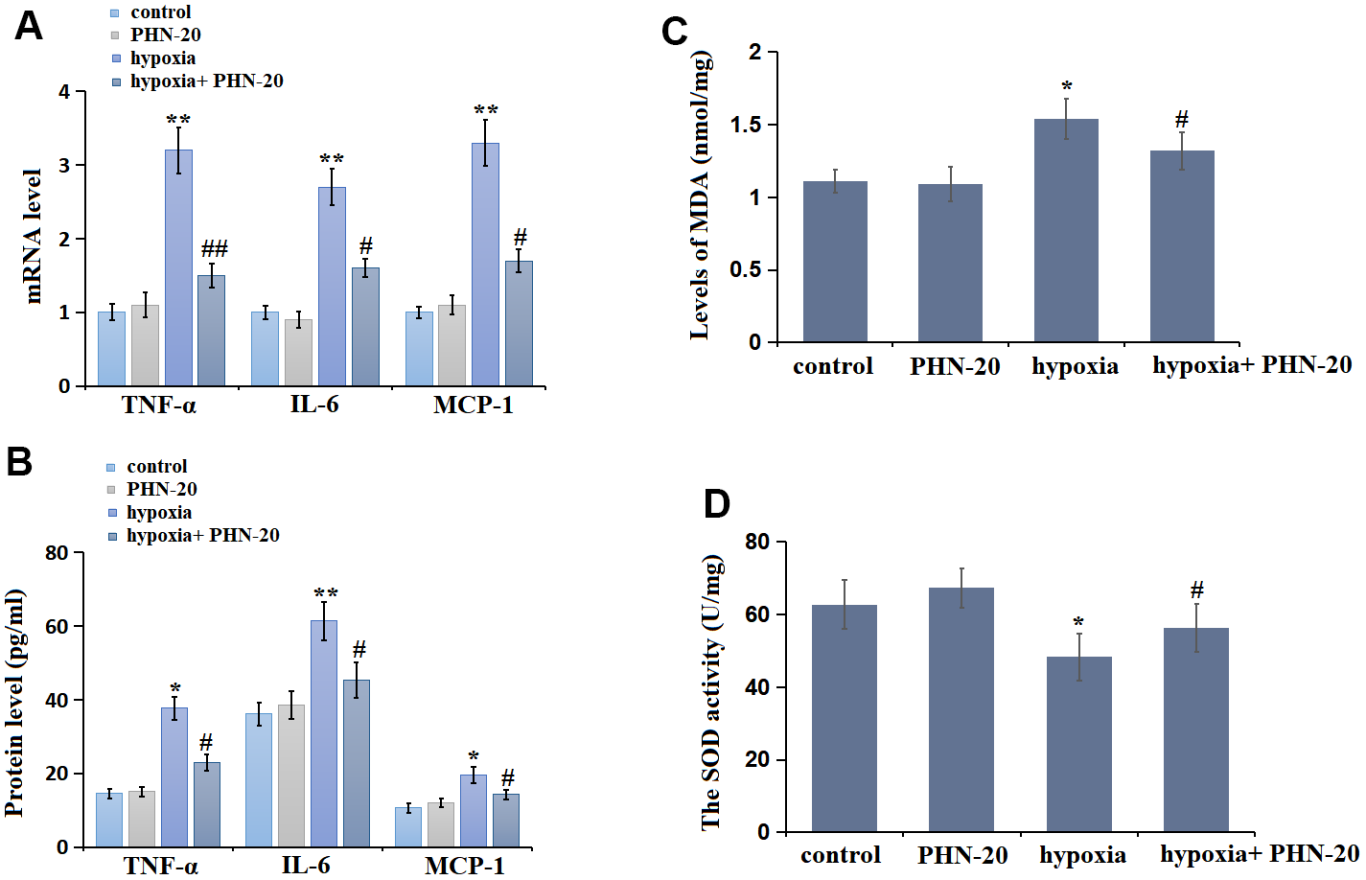
**PHN-20 mitigated inflammatory response and oxidative stress in lung tissues of pulmonary arterial hypertension (PAH) rats.** (**A**) mRNA levels of TNF-α, IL-6, and MCP-1; (**B**) Protein of TNF-α (pg/ml), IL-6 (pg/ml), and MCP-1 (pg/ml) was measured by ELISA; (**C**) The levels of MDA (nmol/mg protein) in lung tissues were measured using a commercial kit; (**D**) The SOD activity (U/mg protein) in lung tissues was measured using a commercial kit. (**, P<0.01 vs. control group; ##, P<0.01 vs. hypoxia group).

The inflammatory state in lung tissues was subsequently evaluated. The levels of tumor necrosis factor –α (TNF-α), interleukin-6 (IL-6), and monocyte chemoattractant protein-1 (MCP-1) were slightly changed in the PHN-20 group, were largely elevated by hypoxia, then signally repressed by PHN-20 (Figure 3A). The protein levels of TNF-α (Figure 3B) in the control, PHN-20, hypoxia, and hypoxia+ PHN-20 groups were 14.52, 15.02, 37.66, and 22.96 pg/mL, respectively. The IL-6 protein level was kept around 37 pg/ml in the control and PHN-20 groups were markedly increased to 61.36 pg/mL in the hypoxia group, then signally reduced to 45.32 pg/mL by PHN-20. Moreover, the protein levels of MCP-1 in the control, PHN-20, hypoxia, and hypoxia+ PHN-20 groups were 10.61, 11.96, 19.60, and 14.25 pg/mL, respectively. The inhibitory effect of PHN-20 on inflammatory response in PAH rats was observed.

### PHN-20 attenuated OS in lung tissues of PAH rats

MDA level and SOD activity are two classic biomarkers of OS. In lung tissues, the MDA level was slightly changed from 1.11 to 1.09 nmol/mg protein in the PHN-20 group, was markedly increased to 1.54 nmol/mg protein in the hypoxia group, then largely reduced to 1.32 nmol/mg protein by PHN-20 (Figure 3C). Furthermore, the SOD activity in the control, PHN-20, hypoxia, and hypoxia+ PHN-20 groups was 62.7, 67.3, 48.3, and 56.4 U/mg protein, respectively (Figure 3D). The repressive effect of PHN-20 on OS in PAH rats was observed.

### PHN-20 inhibited the NLRP3 signaling in lung tissues of PAH rats

**Figure 4:**
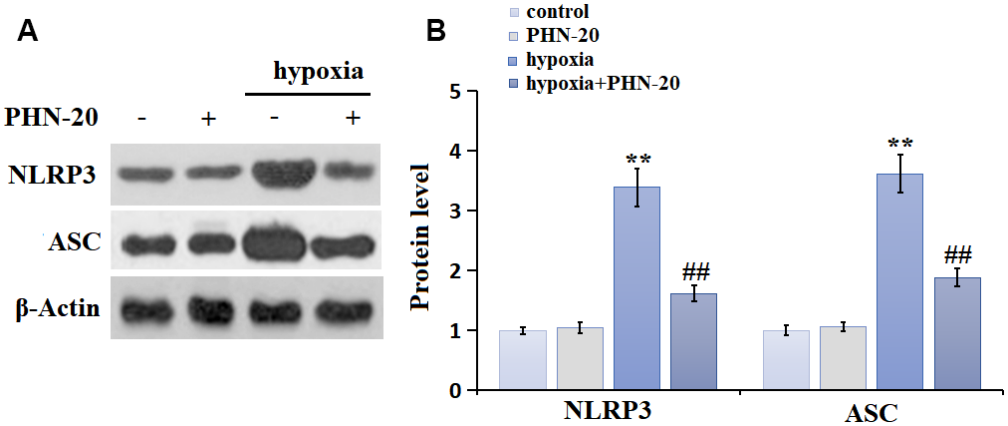
**PHN-20 inhibited the NLRP3 signaling in lung tissues of PAH rats.** (**A**) Protein level of NLRP3. (**B**) Protein level of ASC. (**, P<0.01 vs. control group; #, ##, P<0.05, 0.01 vs. hypoxia group).

NLRP3 signaling is a critical pathway involved in regulating inflammation and OS [13]. The NLRP3 and ASC levels (Figure 4A, 4B) in the PHN-20 group were slightly altered, were largely increased in the hypoxia group then markedly inhibited by PHN-20, suggesting a suppressive property of PHN-20 on NLRP3 signaling in PAH rats.

### PHN-20 repressed inflammation in hypoxia-treated PMECs

**Figure 5:**
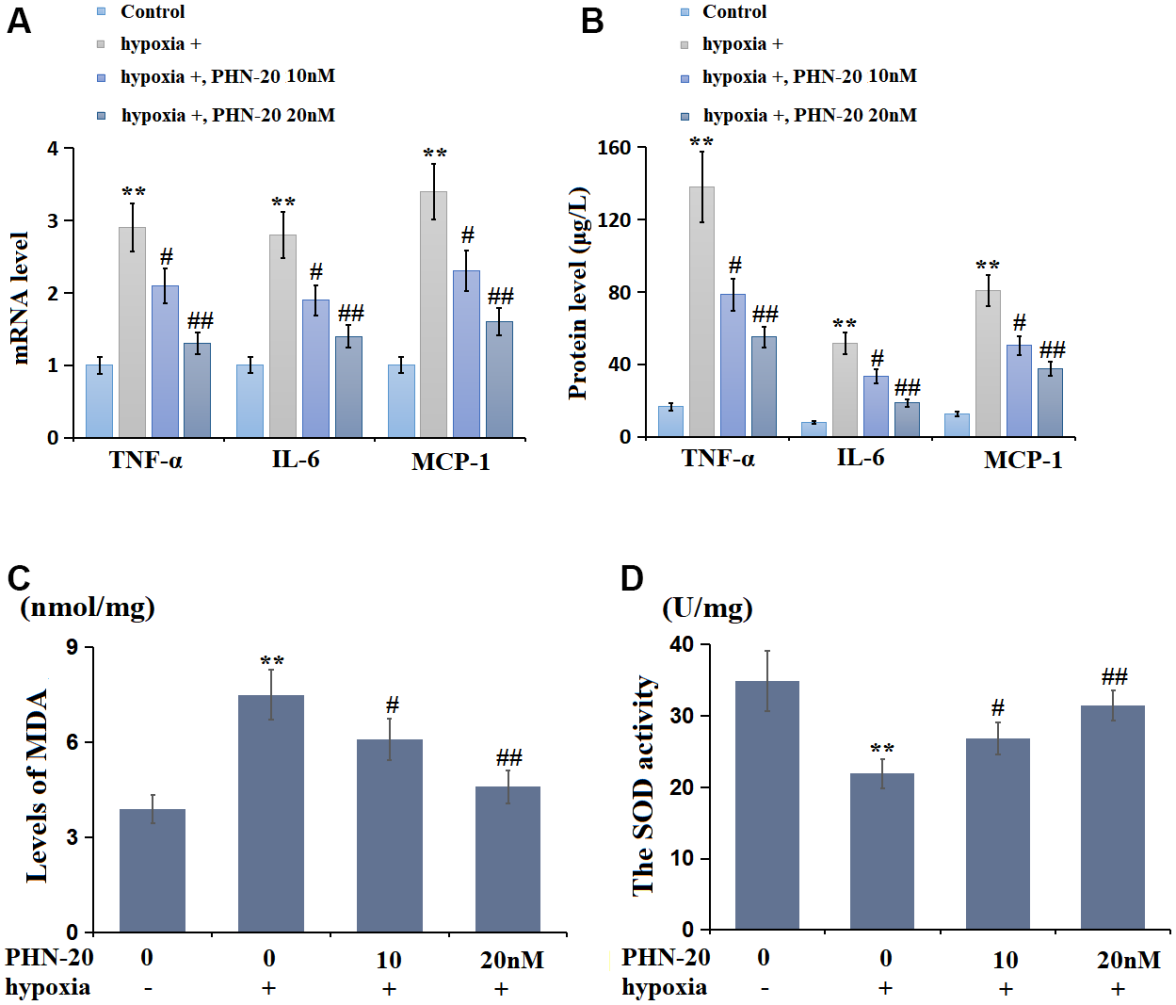
**PHN-20 mitigated inflammatory response and oxidative stress in hypoxia-treated PMECs.** (**A**) mRNA levels of TNF-α, IL-6, and MCP-1; (**B**) Protein levels of TNF-α (μg/L), IL-6 (μg/L), and MCP-1 (μg/L) were measured by ELISA; (**C**) The levels of MDA (nmol/mg protein) were measured using a commercial kit; (**D**) The SOD activity (U/mg protein) was measured using a commercial kit (**, P<0.01 vs. control group; #, ##, P<0.05, 0.01 vs. hypoxia group).

PMECs were treated with hypoxia with or without PHN-20 (10 and 20 nM). The levels of TNF-α, IL-6, and MCP-1 were markedly increased in hypoxia-stimulated PMECs but signally reduced by 10 and 20 nM PHN-20 (Figure 5A). Furthermore, the protein level of TNF-α was largely increased from 16.6 to 138.0 μg/L by hypoxia, then notably decreased to 78.5 and 55.1 μg/L by 10 and 20 nM PHN-20, respectively (Figure 5B). The IL-6 protein levels in the control, hypoxia, 10 nM PHN-20, and 20 nM PHN-20 groups were 7.8, 51.6, 33.5, and 18.6 μg/L, respectively. Moreover, the protein levels of MCP-1 were memorably elevated from 12.4 to 80.8 μg/L by hypoxia but notably decreased to 50.4 and 37.5 μg/L by 10 and 20 nM PHN-20, respectively. The inhibitory effect of PHN-20 on inflammatory response in hypoxia-treated PMECs was observed.

### PHN-20 mitigated OS in hypoxia-treated PMECs

The OS state in PMECs was subsequently evaluated. The MDA levels in the control, hypoxia, 10 nM PHN-20, and 20 nM PHN-20 groups were 3.9, 7.5, 6.1, and 4.6 nmol/mg protein, respectively (Figure 5C). Moreover, the SOD activity was memorably decreased from 34.9 to 21.9 U/mg protein by hypoxia, then prominently repressed to 26.8 and 31.5 U/mg protein by 10 and 20 nM PHN-20, respectively (Figure 5D). The repressive effect of PHN-20 on OS in hypoxia-treated PMECs was observed.

### PHN-20 inhibited the NLRP3 signaling and increased SIRT1 expression in hypoxia-treated PMECs

**Figure 6:**
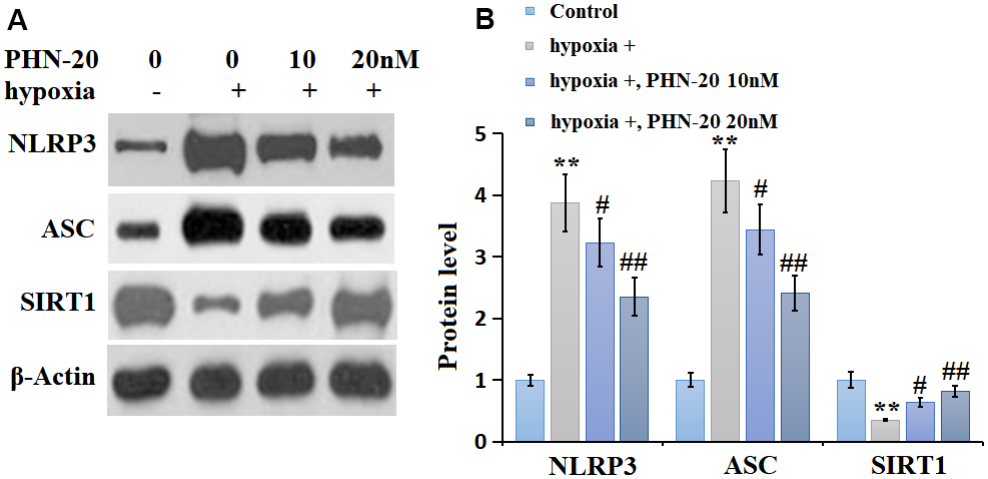
**PHN-20 inhibited the NLRP3 signaling and increased SIRT1 expression in hypoxia-treated PMECs.** (**A**) Western blots of NLRP3, ASC, and SIRT1; (**B**) Analysis of the blots. (**, P<0.01 vs. control group; #, ##, P<0.05, 0.01 vs. hypoxia group).

The levels of NLRP3 and ASC in hypoxia-treated PMECs were signally increased by hypoxia but markedly inhibited by 10 and 20 nM PHN-20. SIRT1 is a regulatory transcriptional factor involved in mediating NLRP3 signaling [14]. SIRT1 was markedly downregulated in hypoxia-treated PMECs but largely reversed by 10 and 20 nM PHN-20 (Figure 6), implying a correlation might exist between the function of PHN-20 and SIRT1.

### Silencing of SIRT1 abolished the inhibitory effect of PHN-20 on NLRP3 signaling in hypoxia-treated PMECs

**Figure 7:**
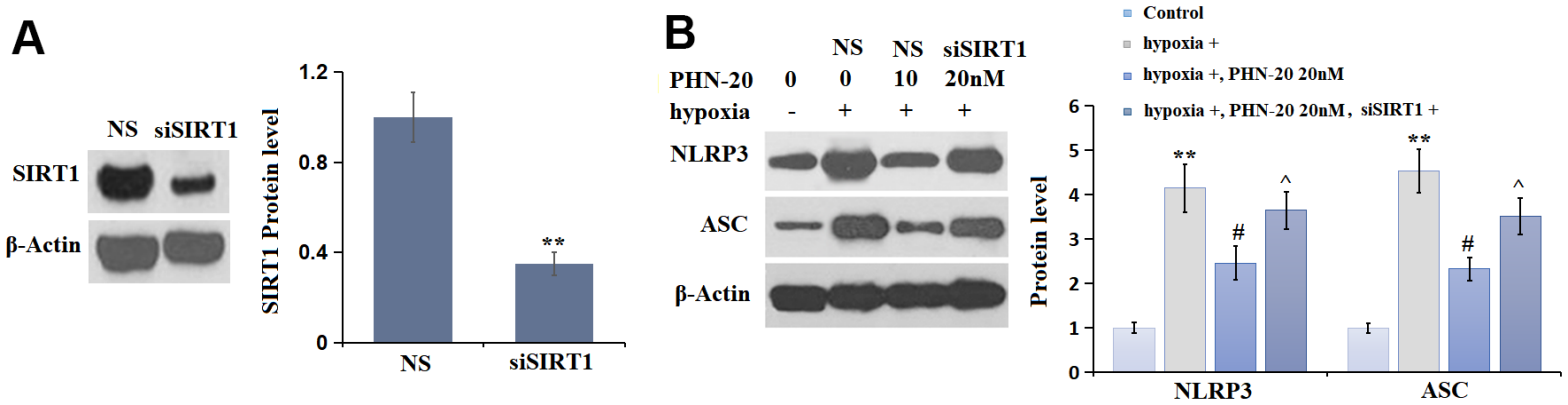
**Silencing of SIRT1 abolished the inhibitory effects of PHN-20 on NLRP3 signaling in hypoxia-treated PMECs.** PMECs were transduced with lenti-viral SIRT1 shRNA, followed by stimulation with hypoxia in the presence or absence of PHN-20 (20 nM) for 24 hours. (**A**) Western blot revealed successful knockdown of SIRT1; (**B**) Western blots of NLRP3, ASC. Protein levels of NLRP3 and ASC. (**, P<0.01 vs. control group; #, P<0.05 vs. hypoxia group; ^, P<0.05 vs. PHN-20 group).

To verify the correlation, PMECs were transduced with lenti-viral SIRT1 shRNA, followed by stimulation with hypoxia, with or without PHN-20 (20 nM) for 24 hours. The efficacy of SIRT1 silencing was identified by Western blot (Figure 7A). The largely increased NLRP3 and ASC levels observed in hypoxia-treated PMECs were repressed by PHN-20, which were notably reversed by the knockdown of SIRT1 (Figure 7B). The regulatory function of PHN-20 on NLRP3 signaling in hypoxia-treated PMECs was mediated by SIRT1.

## DISCUSSION

Vascular remodeling is the most important pathological change of PAH, which is found to be regulated by IL-6, including perivascular T lymphocyte recruitment, stimulating endothelial cells to produce cytokines, and promoting the proliferation of pulmonary artery smooth muscle and pulmonary vascular endothelial cells [15]. By applying a monoclonal anti-IL-6 receptor antibody, TAKAHIRO et al. [16] found that hypoxia-induced PAH was improved and the accumulation of Th17 cells and M2 macrophages in lung tissues was repressed. In addition, KWON et al. [17] showed that the TNF-α antagonist exerted a weak protective effect on PAH by inducing changes in pulmonary vessels and affecting the expression of inflammatory cytokine genes related to PAH. In our study, high secretion of IL-6 and TNF-α was observed in PAH rats, which was identified by elevated values of RVSP, mPAP, RV/LV + S, and W%. Furthermore, enlarged production of IL-6 and TNF-α was also observed in hypoxia-stimulated PMECs. These symptoms were in line with the results reported by Liu [18] and Pan [19]. Following the administration of PHN-20, markedly declined values of RVSP, mPAP, RV/LV + S, and W% were observed, accompanied by a decreased production of cytokines in lung tissues and hypoxia-stimulated PMECs, implying that the alleviative effect of PHN-20 against PAH might be associated with its anti-inflammatory property.

Pulmonary artery endothelial injury is one of the important mechanisms leading to vascular remodeling in PAH [20]. The pulmonary vascular endothelium is located between blood and tissue with the ability to secrete a variety of vasoactive substances, such as endothelin-1, angiotensin II, and vascular endothelial growth factor. Under physiological conditions, the levels of these substances are in equilibrium to maintain normal vascular tension, regulate the proliferation and migration of vascular smooth muscle cells, and mediate platelet adhesion [21]. Wunderlich et al. [22] found that the normal function of eNOS in vascular endothelial cells is regulated by Caveolin-1. In Caveolin-1 knockout mice, loss of eNOS regulation leads to Enos uncoupling, resulting in the flow of electrons to oxygen molecules and subsequent secretion of ROS. Increased ROS further reduces the synthesis of NO by inhibiting eNOS activity to aggravate vascular endothelial OS injury, which promotes the proliferation and migration of vascular smooth muscle cells and accelerates vascular remodeling. In our study, OS was found activated in both lung tissues of PAH rats and hypoxia-stimulated PMECs, which was consistent with data presented by Wang [23] and Yu [24]. After PHN-20 treatment, OS was signally repressed in both lung tissues of PAH rats and hypoxia-stimulated PMECs, implying that the alleviative effect of PHN-20 against PAH might be correlated with the inhibition of OS.

NLRP3 is a receptor in innate immune cells, that recognizes a variety of endogenous danger signals, such as ROS and ATP, and exogenous danger signals, such as uric acid crystals and aluminum hydroxide. In general, NLRP3 is in a self-inhibitory state. When NLRP3 is activated by a variety of danger signals, the production of the coupling head protein ASC and activated Caspases-1 will be induced to form a complex protein enzyme complex, namely NLRP3 inflammasome, in which activated Caspase-1 promotes the secretion and processing of inflammatory cytokines [25, 26]. Recently, NLRP3-mediated OS and inflammation have been found in PAH rats [27]. In our study, in line with reports of Mavrogiannis [28] and Zhou [29], activated NLRP3 signaling was observed in both PAH rats and hypoxia-stimulated PMECs, which were markedly abolished by PHN-20 administration, implying the involvement of NLRP3 inhibition in PHN-20 function against PAH. SIRT1 is an inhibitory transcriptional factor of NLRP3 signaling [30], which was found notably downregulated in hypoxia-stimulated PMECs in our study. After PHN-20 administration, the SIRT1 level in PMECs was found markedly increased. Furthermore, the suppressive property of PHN-20 on the NLRP3 pathway in hypoxia-stimulated PMECs was abrogated by SIRT1 knockdown, suggesting that the function of PHN-20 in PAH might be mediated by SIRT1. In future work, the specific target of PHN-20 in PMECs for the regulation of SIRT1 will be further confirmed to deeply understand the functional mechanism of PHN-20 in PAH.

In this study, we assessed the effects of PHN-20 on oxidative stress and inflammatory response in PAH. However, PAH remains a severe clinical condition and the PAH disease process is complex to the extent that the current findings may not completely imitate the pathological mechanisms of PAH. A diversity of risk factors is reported to be involved in PAH, including genetics, ageing, and obesity [31]. In the past decades, PAH-specific therapies have been developed targeting the prostacyclin, endothelin, and nitric oxide pathways [32]. The recent advancement has been in developing strategies targeting novel pathways. Therefore, we should further verify the interaction of related proteins and the upstream as well as the downstream signaling molecules. In the future, we will design more experiments both *in vivo* and *in vitro* to reveal the molecular mechanisms underlying PHN-20 exerts its protective actions in PAH.

In conclusion, PHN-20 ameliorated PAH via repressing OS and inflammation by mediating the transcriptional function of SIRT1. The findings of our study provide a scientific basis for the promising use of PHN-20 as therapeutics in the clinical treatment of PAH.

## MATERIALS AND METHODS

### PAH modeling and grouping

Twenty-four 7-9 week male rats were obtained from Vital River. They were divided into four groups: Control, PHN-20, hypoxia, and hypoxia+ PHN-20. In the Control and PHN-20, normal rats were injected with normal saline and 100 ng/g/day PHN-20 for 28 days, respectively. After one week of pre-adaptation to the laboratory environment, rats in the hypoxia groups were placed in the hypoxia chamber to simulate the altitude of 5500 m plateau hypoxia environment (55KPa air pressure and 10.5% oxygen concentration environment) for continuous hypoxia, with 12 h lighting per day, the temperature controlled at 20 to 22° C, and the hypoxic chamber opened every 2 to 3 hours. Within 1 hour, cages were cleaned and supplemented with food and water, and food was consumed with AD libitum. In the hypoxia and hypoxia+ PHN-20 groups, hypoxia-treated rats were injected with normal saline and 100 ng/g/day PHN-20 for 28 days, respectively.

### The measurement of pulmonary hemodynamic parameters

Rats in each group were anesthetized by intraperitoneal injection of 12% urethane (1000 mg/kg) before operation. The polystyrene microcatheter was connected to the BL-420E biological function experimental system with a pressure transducer. The value of mPAP was measured, the catheter was slowly withdrawn, and the value of RVSP was measured.

### The determination of RV/LV + S

The right ventricle (RV) and left ventricular septum (LV+S) were separated along the ventricular septum, and the surface blood was washed with normal saline. Subsequently, the filter paper was used for blotting samples and then weighted using an electronic balance. The value was calculated as RV/LV + S.

### Enzyme-linked immunosorbent assay (ELISA)

Different concentrations of standards were added to the standard wells and testing samples were loaded in the sample well, followed by introducing 40 μL sample diluent. Horseradish peroxidase (HRP) labeled antibody was added to each well of the standard sample well and the sample well was sealed and incubated at 37° C for 60 min in the incubator. After removing the liquid using an absorbent paper, the washing liquid was added for washing and repeated 5 times. Substrates A and B were then added to each reaction well and incubated for 15 min at 37° C in the dark. The OD value of each well was determined with a microplate reader (BioTek, USA) within 15 min after 50 μL of termination solution was added.

### The measurement of MDA level and SOD activity

A commercial kit (Qingdao Jisskang Biotechnology, China) was utilized for the detection of MDA level using the TBA method, while the SOD activity was tested with a commercial kit (Beyotime, China) using the nitroblue tetrazolium (NBT) method. The instructions of the kits were strictly followed.

### Real-time PCR assay

RNAs were extracted from cells or tissues using the Trizol reagent and then transcribed into cDNA with the reverse transcription kit (MedChemExpress, USA). Subsequently, the PCR reaction was conducted with the 2×SYBR Green PCR Master Mix kit (Lifeint, China). The gene level was determined utilizing the 2^−ΔΔCt^ method.

### Western blotting assay

Total proteins were extracted and quantified with the BCA method, which was conducted with the separation using the 12% SDS-PAGE, followed by being transferred to the PVDF membrane. Following blocking, primary antibodies against NLRP3, ASC, SIRT1, and β-actin (1:500, Bosterbio, USA) were added to be cultured for 12 h at 4° C, followed by incubation with the secondary antibody (1:1000, Bosterbio, USA) and incubated for 60 min. Lastly, the ECL solution was added for exposure and the expression level was quantified with the Image J software.

### Cells and treatments

Human PMECs were purchased from Science Cell (USA) and were grown in an endothelial medium supplemented with 10% FBS. The incubation condition was maintained at 37° C and 5% CO_2_. To achieve SIRT1-knockdown PMECs, cells were transduced with lenti-viral SIRT1 shRNA (Genscript, China) for 48 h, followed by evaluating the efficacy with the Western blotting assay.

### Statistical analysis

Data were expressed using mean± standard deviation (S.D.) and analyzed using the GraphPad prism software 6.0. The data were compared using a single-factor analysis of variance (ANOVA) following the determination of variance. Comparisons among multiple groups were assessed using the Least Significant Difference method. If the variance was not homogeneous, Tamhane’s multiple comparison procedure was used to assess comparisons among multiple groups. P-values <0.05 were considered significant.
